# The Use of Poly(*N*-vinyl pyrrolidone) in the Delivery of Drugs: A Review

**DOI:** 10.3390/polym12051114

**Published:** 2020-05-13

**Authors:** Paola Franco, Iolanda De Marco

**Affiliations:** Department of Industrial Engineering, University of Salerno, Via Giovanni Paolo II, 132, 84084 Fisciano (SA), Italy; pfranco@unisa.it

**Keywords:** polyvinylpyrrolidone, drug delivery systems, microparticles, nanoparticles, fibers, hydrogels, tablets, films

## Abstract

Polyvinylpyrrolidone (PVP) is a hydrophilic polymer widely employed as a carrier in the pharmaceutical, biomedical, and nutraceutical fields. Up to now, several PVP-based systems have been developed to deliver different active principles, of both natural and synthetic origin. Various formulations and morphologies have been proposed using PVP, including microparticles and nanoparticles, fibers, hydrogels, tablets, and films. Its versatility and peculiar properties make PVP one of the most suitable and promising polymers for the development of new pharmaceutical forms. This review highlights the role of PVP in drug delivery, focusing on the different morphologies proposed for different polymer/active compound formulations. It also provides detailed information on active principles and used technologies, optimized process parameters, advantages, disadvantages, and final applications.

## 1. Introduction

Polyvinylpyrrolidone (PVP), also called polyvidone or povidone, is a biodegradable, water-soluble polymer, derived from its monomer *N*-vinylpyrrolidone. In addition to being a hydrophilic polymer, PVP has excellent solubility in solvents of different polarities, good binding properties, and a stabilizing effect for suspensions and emulsions [1]. PVP is a biocompatible and non-toxic polymer; indeed, it was also recognized as safe by the Food and Drug Administration (FDA). For this reason, in addition to the food sector, PVP is widely used in medicine and cosmetics, for pharmaceutical and biomedical applications [2,3,4,5,6]. PVP has unique physical and chemical features; e.g., it is essentially chemically inert, colorless, temperature-resistant, and pH-stable. The various molecular weight PVPs are distinguished by different K-values, e.g., K12 (3100–5700 Daltons), K17 (7900–10,800 Daltons), K25 (23,000–32,000 Daltons), K30 (35,000–51,000), and K90 (900,000–1,300,000 Daltons) [7].

Up to now, PVP has been used in the pharmaceutical and biomedical fields to develop different drug delivery systems, such as oral, topical, transdermal, and ocular administration. Besides, PVP is also useful in the delivery of genes [8,9,10,11,12] or can be coupled with metal particles for regenerative medicine [13,14,15] and targeted-delivery [16,17]. Therefore, PVP results to also be a very versatile polymer. Indeed, different morphologies using PVP as the polymeric carrier have been proposed for drug delivery. PVP allows obtaining a drug-controlled release, improving the bioavailability of poorly water-soluble drugs, protecting the active compound against external agents (pH, temperature, and oxygen), and masking unpleasant odors and flavors. Many active compounds belonging to different categories have been incorporated into PVP microparticles and nanoparticles. The PVP-based particles have been produced with different technologies, from the most traditional (for example, spray drying) to the more innovative ones (for example, supercritical fluids-assisted techniques) [2,18,19,20,21]. Being a well-spinnable polymer, PVP fibers loaded with various active ingredients have also been produced [4,22,23,24,25]. PVP-based hydrogels [5,26,27,28,29] and oral tablets [27,30,31,32] have also been proposed in some papers. Furthermore, PVP exhibits an excellent ability to form films [6,33,34,35,36].

The versatility and the unique properties of PVP make it a polymer with great potential for the production of pharmaceutical formulations. Indeed, the use of PVP in the emerging pharmaceutical and biomedical areas can positively contribute to offering solutions still required by the pharmaceutical market. Despite this, in the literature, there is no systematic organization of the numerous studies conducted until now using PVP as a carrier.

This review focuses on the use of PVP in drug delivery. In particular, the obtained results are classified according to the morphology of the proposed pharmaceutical formulations. Selected active principles, employed methods, potential applications, advantages, and disadvantages are highlighted. Hence, for each morphology/system selected, important information about the achievements to date, the most effective methods, and possible future perspectives are available in this review.

## 2. Different Morphologies as Drug Delivery Systems

### 2.1. Microparticles and Nanoparticles

**Figure 1:**
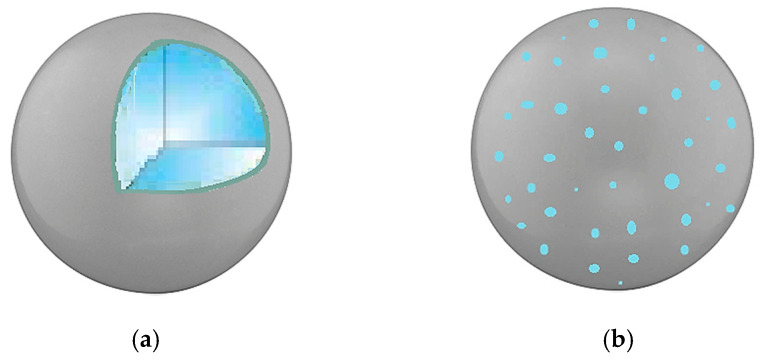
Possible PVP-based particles: (**a**) core–PVP shell structure (microcapsule-like structure); and (**b**) active principle dispersed into PVP matrix (microsphere-like structure).

The production of microparticles and nanoparticles offers unique benefits in drug delivery. Size reduction is a common approach to overcome the problems related to poor solubility in water of many active ingredients, such as low oral bioavailability, irregular absorption, and side effects caused by drug overdose. The drug dissolution rate can be enhanced by its micronization since the particle size reduction leads to an increase in the specific surface area and a higher contact between the drug molecules and the dissolution medium [37]. However, this route does not allow stabilizing the pharmaceutical formulations. For this purpose, several hydrophilic polymers were used as carriers to obtain polymer/drug particles. Among these, PVP is one of the most commonly used for the attainment of composite particles [2,19,38,39,40,41,42,43,44,45]. The presence of PVP allows the delay of the crystallization of the active principle contained in the particles and the increase of its dissolution rate in aqueous media. Moreover, PVP is used as a masking agent for odors/flavors, as well as a stabilizing or protective agent for the active compound, avoiding its oxidation and deactivation. PVP was widely used to obtain both microcapsules [19,40] and microspheres [2,21,39,42,46,47,48,49,50,51,52,53,54,55,56]. A core–shell structure characterizes the microcapsules (Figure 1a); generally, the core material contains the active compound, whereas the wall material consists of the polymeric carrier (PVP or blends). Differently, a microsphere (Figure 1b) consists of a matrix (PVP or polymeric blends) in which the drug is homogeneously dispersed. In some papers, composite nanoparticles were also produced by using PVP as the carrier [17,57].

As shown in some papers [16,17,58,59], PVP-based nanoparticles can be exploited for the targeted drug delivery, to kill tumor cells selectively. In the work of Rose et al. [17], magnetite (Fe_3_O_4_) nanoparticles were coated with PVP to assure a magnetically targeted drug delivery. Then, Epirubicin hydrochloride, chosen as the model anticancer drug, was adsorbed onto the surface of PVP-coated and uncoated nanoparticles. It was demonstrated that the PVP coating reduced the agglomeration of nanoparticles; besides, PVP led to a slightly higher drug loading (78%), compared to the uncoated nanoparticles. The cancer-targeting ability of PVP-coated nanoparticles was ascertained since PVP-coated nanoparticles showed 81% of growth inhibition in breast cancer cells. Similarly, in another work [16], gold nanoparticles coated with PVP (mean size about 14 nm) were applied for the targeted delivery of Doxorubicin. In particular, the drug-loaded PVP/gold nanoparticles inhibited the growth of lung cancer cells more effectively than unprocessed Doxorubicin and drug-unloaded nanoparticles.

Up to now, PVP-based microparticles were prepared by different techniques; among the most common processes, there is spray drying [2,3,18,38,39,60,61] consisting of the atomization of a liquid solution through an injector and a hot gas flow drying, which favors the drying of the droplets. For example, Bothiraja et al. [2] produced spherical PVP K30 microparticles containing andrographolide, a bitter lipophilic compound with numerous benefits, including analgesic, antipyretic, anti-inflammatory, antithrombotic, anticancer, and hypoglycaemic activities. Amorphous particles at different PVP/andrographolide ratios, namely 4/1, 3/1, and 2/1 w/w, were obtained. The composite microparticles exhibited better flow properties than those of the pure drug and those of the polymer/drug physical mixture. The entrapment of the active principle into the amorphous PVP matrix inhibited the drug’s recrystallization, as also highlighted in another paper [39]. In particular, the stability of microparticles was proved for three months. The high therapeutic efficacy of composites was highlighted from in vivo tests.

Additionally, the composite microparticles showed a five-fold increase in andrographolide dissolution compared to that of the pure compound and that of the physical mixture. Moreover, the release rate enhanced by increasing the PVP/drug ratio. A similar effect of PVP/drug ratio on the dissolution of the active compound from particles was also observed in other studies [38,39,43,46,47,50,62], in which both spray drying and other micronization technologies were employed. In some cases, a low PVP content in the spray-dried particles did not lead to any improvement in the dissolution of the active compound [38,39], as also occurred with other techniques [45,50]. Generally speaking, it has been demonstrated that the morphology of drug-loaded PVP particles obtained through spray drying is strongly influenced by different parameters, mainly the gas flow rate or the inlet and outlet temperatures. These parameters also affect the formulation stability, the drug recrystallization kinetics, and the drug dissolution [3,60]. For example, Paudel et al. [60] studied the influence of the inlet temperature (in the range 40–120° C) and the air flow rate (in the range 5–15 L/min) on the properties of the PVP/naproxen particles 3/2 *w*/*w*. The amorphous particles prepared at a higher inlet temperature and a greater airflow rate led to a faster drug dissolution; moreover, they were more stable in the presence of moisture, considering that the drug recrystallization was slower. Moreover, the highest solvent residues were found at the mildest solvent evaporation conditions; i.e., the lowest inlet temperature and airflow rate.

Other conventional techniques were used to obtain drug-loaded PVP particles, including freeze-drying [63], coacervation [19,40], and co-grinding [57,64]. In particular, in the work of Cavallari et al. [63], indomethacin was co-freeze-dried with PVP; then, PVP/drug powders were encapsulated into molten stearic acid using ultrasonic spray-congealing. Scanning Electron Microscopy (SEM) revealed that the freeze-dried PVP/indomethacin system showed reduced size compared to both unprocessed materials; however, the morphology was still crystalline. Rough microcapsules were instead obtained with the addition of stearic acid. However, as proved by X-Ray Diffraction (XRD) analysis, samples still showed a crystalline structure, especially after freeze-drying, maintained at least in part in the final PVP/stearic acid/drug capsules. Although the structure of the samples was not completely amorphous, an improvement in the drug dissolution was observed. Indeed, 20% of pure drug dissolved after 70 h, whereas 70% of the drug was released after 10 min and 20 min from PVP/stearic acid capsules and freeze-dried PVP/drug powders, respectively.

As mentioned, the co-grinding method was also used to obtain PVP-based solid dispersions. Mennini et al. [64] developed a new multi-particulate system, which consisted of chitosan/calcium chloride (CaCl_2_)/alginate microspheres for the colon-targeted delivery of celecoxib/hydroxypropyl-β -cyclodextrin (HP-β-CD)/PVP complexes. In particular, complexes were prepared by co-grinding using both the binary system celecoxib/HP-β-CD (1/1 mol/mol) and the ternary system drug/HP-β-CD/PVP (adding 10% *w*/*w* of PVP). The dissolution studies showed that the release of celecoxib from the ternary drug/HP-β-CD/PVP co-ground system was two and three times faster than the binary drug/HP-β-CD co-ground system and the pure drug, respectively. These results are in agreement with other studies [65,66]. Therefore, the addition of PVP allowed to improve further the dissolution rate of the active principle compared to the drug/cyclodextrin inclusion complexes. Indeed, Valero et al. [67] highlighted that the presence of PVP influenced the interactions between the drug and the cyclodextrin and even acted as a bridge between the cyclodextrin molecules.

Instead, the coacervation method has been revealed to be very promising to encapsulate active compounds in PVP-based microcapsules, although toxic organic solvents are widely employed for this purpose [19,40]. The encapsulation allowed protecting the active core material from thermal, chemical, or mechanical degradation, as well as modifying its dissolution rate. It was demonstrated that several factors influenced the release kinetics of active compounds from microcapsules prepared using PVP as shell-former. Among these factors, there are the dissolution medium pH, the shell thickness, the molecular weight of PVP, or that of the active compound, as well as the use of copolymers or polymeric blends to form the shell [19,40]. Dowding et al. [19] demonstrated that the release rate of the encapsulated active compound decreased by increasing the PVP shell thickness or the PVP molecular weight (due to the formation of stronger interactions between low molecular weight PVP and the model drug). Moreover, pH-responsive microcapsules can be obtained, thanks to an increase in the drug release rate by decreasing the pH of the dissolution medium, because of the PVP swelling at low pH value [19,40].

However, some parameters play a key role in obtaining microcapsules by coacervation [40]. In particular, Gun and Routh [40] proved that a fast evaporation rate, which was obtained at the temperature of 40 °C, led to the formation of incomplete PVP shells or even the precipitation of PVP granules. In contrast, a slow evaporation rate (obtained when the temperature was fixed at 20 °C) allowed forming microcapsules with complete PVP shells, as observed from SEM images. These results were in agreement with the dissolution tests. Indeed, the dissolution time of the active compound from the samples obtained with a fast evaporation rate was similar to that obtained without PVP at all. On the contrary, a slower release was reached with capsules produced at a slow evaporation rate, because the complete shells provided a barrier to the drug release.

In recent years, among the new strategies, the multilayer encapsulation of active compounds, including poorly-water soluble drugs, stands out to fabricate layer-by-layer capsules. The layer-by-layer assembly of multilayer films onto particles, followed by the selective removal of the sacrificial template, offers many advantages. These include good control of the size, shape, composition, and shell thickness of the obtained capsules, in addition to a flexible geometry, a tunable shell permeability, and high drug loadings [68,69]. This method also allows producing capsules that respond to external stimuli, such as pH, chemicals, and physical agents (e.g., light, magnetic field, and ultrasound). In general, the active molecules can be incorporated in layer-by-layer capsules using two main approaches: pre-loading or post-loading [69]. The drug pre-loading can be obtained by two different routes: the active molecules can be pre-loaded in a porous inorganic template, followed by the polymeric coating; alternatively, the previous fabrication of microparticles containing the drug is followed by the coating with polymers. In the post-loading approach, drastic changes of medium conditions (e.g., pH, ionic strength, temperature, etc.) are exploited to increase significantly the shell permeability, thus the active molecules are able to penetrate the polymer network driven by the concentration gradient. The main issue of the post-loading is that the required changes of the medium conditions may lead to the drug deactivation.

PVP was employed to produce layer-by-layer capsules in few works [70,71,72,73]. Zhang et al. [70] fabricated hollow capsules through the layer-by-layer method. Two spherical templates, namely polystyrene and silica particles, were sequentially coated with PVP and m-methylphenol-formaldehyde resin (MPR). PVP and MPR were employed as the hydrogen acceptor and donor, respectively, leading to the formation of hydrogen bonds. Then, the sacrificial core was removed to obtain the hollow particles. However, intact capsules were produced using silica as the template, whereas the removal of polystyrene led to the shell collapse.

Moreover, from the transmission electron microscopy (TEM) images, it was observed that the roughness of the particle surface increased as the number of PVP/MPR bilayers increased. However, it was noted that PVP/MPR capsules tended to form aggregates. Therefore, a polyelectrolyte bilayer consisted of poly(acrylic acid) (PAA) and poly(allylamine hydrochloride) (PAH) was added to the capsules. The addition of the PAA/PAH bilayer stabilized the capsules, which exhibited a reduced tendency to aggregation due to the charge on the capsule surface. The authors proposed the obtained hollow capsules for medicine and pharmaceutical applications.

Similarly, Kumar et al. [71] used silica particles (4.27 ± 0.23 μm) as a sacrificial template to prepare layer-by-layer capsules. Multilayer capsules based on PVP and poly(methacrylic acid) (PMA) were proposed to encapsulate rifampicin, an anti-tuberculosis drug. The removal of silica core through a buffer of ammonium fluoride and hydrofluoric acid at pH 3 allowed producing better capsules with respect to the use of hydrofluoric acid only. Most of the obtained capsules exhibited eight layers, with an overall shell thickness of 20 ± 0.4 nm. The drug was post-loaded. The dissolution tests showed a burst-like release at pH values higher than 7. Indeed, in these conditions, the layer-by-layer microcapsules were rapidly disintegrated, thus releasing the active principle in the short term. The microbial interaction studies with *Mycobacterium smegmatis* proved the preservation of the activity of the encapsulated drug. Instead, Dam and Caruso [72] succeeded in stabilizing polyrotaxane capsules, previously obtained using silica particles as a template, by adding a stabilizing outer bilayer of PVP/PMA. Using the layer-by-layer assembly, Parekh et al. [73] produced nanocapsules (size of about 160 nm) containing camptothecin in 8-bilayered shells, which consisted of heparin and block-copolymer of polylysine and polyethylene glycol (PEG). However, in this study, particular attention was given to the influence of PVP used as an excipient to improve the colloidal stability of the dispersion of drug-loaded nanoparticles. In detail, the concentration of PVP in the medium used for the core preparation was varied from 0 to 2.2 mg/mL. It was noted that, increasing the PVP content in the core formulation, the core dimensions reached a maximum value at 0.7 mg/mL as PVP concentration and, then, decreased significantly.

Despite the advantages, such as the easy scale-up in the case of spray drying, the conventional processes generally used to obtain particles have some critical drawbacks, including solvent residues in the product, high process temperature, and, often, the production of irregular particles [38,39,60,63].

To overcome these limits, supercritical carbon dioxide (CO_2_) assisted technologies were proposed to produce drug-loaded PVP microparticles, mainly Supercritical Assisted Atomization (SAA) [46,47,48,49] and Supercritical AntiSolvent (SAS) process [20,21,41,42,43,44,45,50,51,52,53,54,55,56,74,75,76]. In detail, the CO_2_ acts as a cosolute in the SAA process and as an antisolvent in the SAS process. PVP revealed to be a particularly suitable carrier to be processed by these two supercritical fluid-based techniques. Micrometric and sub-micrometric composite particles with an amorphous structure can be obtained by properly selecting the operating conditions. This allowed significantly increasing the dissolution rate of various active compounds belonging to different categories, such as antioxidants [41,46,47,48,49,53,54,56,74], non-steroidal anti-inflammatory drugs (NSAIDs) [20,51,55,76], vitamins [42,52], and corticosteroids [21]. Indeed, the mild operating conditions used in supercritical CO_2_-based technologies even allow the processing of thermosensitive compounds without degradation or deactivation, e.g., preserving the antioxidant activity of the active material [48,49,56].

In the study of Wu et al. [20], the excellent performance of supercritical fluid assisted processes was demonstrated by comparing spray drying and SAS techniques to produce PVP/piroxicam 4/1 *w*/*w* microspheres. A narrow particle size distribution was obtained by using the SAS process with respect to spray-drying. SAS composites also showed shorter drug dissolution times than spray-dried samples. Indeed, after 5 min, the percentage of piroxicam released in the dissolution medium in the case of the unprocessed drug, of the drug released from spray-dried microparticles, and of the drug released from SAS-processed microparticles was equal to 2%, 55%, and 100%, respectively.

In general, good SAS coprecipitation results are obtained when microspheres are precipitated from the drying of microdroplets, while the coprecipitation occurs partially when nanoparticles or sub-microparticles are obtained [53,77,78]. From the SAS literature, it is clear that different process parameters influence the attainment of PVP-based microparticles. For example, an increase in the operating pressure generally causes a decrease in particle size. In contrast, the increase of the concentration of solutes in the injected liquid solution leads to an increase in the particle dimensions [78]. Instead, regarding the SAA process, the gas-to-liquid ratio (GLR; i.e., the weight ratio between CO_2_ and the liquid solution) strongly influences the particle morphology and dimensions [46,47,48,49]; indeed, by increasing the GLR, the particle dimensions decrease and the particle size distribution shrinks.

Recently, a single-step PVP/curcumin coprecipitation and coating onto different polymers was proposed by Matos et al. [74]. Briefly, a SAS-like coprecipitation was performed using PVP as the carrier, while another powder polymer had previously been charged into the precipitation chamber. In this way, the precipitated PVP/curcumin particles were deposited as to form a coating onto irregular particles/crystals of different polymers; i.e., microcrystalline cellulose (MCC) (size: 175 µm), corn starch (size: 15 µm), or lactose (size: <5 µm). The stirring is guaranteed in the precipitation chamber during the single-step coprecipitation and coating. However, no further significant increase in the drug dissolution rate occurred with coating onto different polymers with respect to the simple SAS PVP/curcumin coprecipitation. Indeed, PVP/curcumin coprecipitated powders already allowed reaching more than 95% of release in 5 min, compared to about 2% of the unprocessed active compound.

Micelle-like particles are a recently proposed novel drug delivery system to improve the bioavailability of poorly water-soluble drugs [79,80,81]. A polymeric micelle has a core–shell structure, which is formed in an aqueous solution. It is prepared with amphiphilic block copolymers, combining hydrophilic and hydrophobic segments. When the concentration of the block copolymers in the aqueous medium is higher than a certain concentration, known as the critical aggregation concentration, the micelles are formed to decrease the free energy. Indeed, at this concentration, the hydrophobic portions of the block copolymers tend to associate in order to minimize the contact with the water molecules. This phenomenon leads to the formation of the typical core–shell structure of micelles. Hence, the inner hydrophobic core of the polymeric micelles can host hydrophobic active compounds.

Polymeric micelles formed by *N*-vinylpyrrolidone copolymers were found to be particularly suitable as amphiphilic carriers to incorporate poorly water-soluble drugs [80,81,82,83,84,85,86,87]. Different amphiphilic derivates, based on hydrophilic PVP with a terminal hydrophobic segment, were prepared to form nanoparticles in aqueous solutions. In particular, Kuskov et al. proposed this amphiphilic PVP nanocarrier to deliver proteins [84] or indomethacin [83]. In both cases, a two-stage method was used to synthesize amphiphilic PVP. Firstly, PVP with one terminal carboxylic group was synthesized by free-radical polymerization of *N*-vinylpyrrolidone. Then, hydrophobic n-alkyl groups were attached to the PVP reactive terminus. The drug-loaded particles were prepared by the solvent evaporation method. Bowman–Birk soybean proteinase inhibitor and its hydrophobized derivatives were selected as model proteins and loaded in polymeric particles of 50–80 nm. The encapsulation in the polymeric micelles preserved the activity of the proteins at low pH values. Instead, a drug sustained-release was reached loading indomethacin (a model NSAID) in micelle-like particles. A narrow particle size distribution was obtained. The particle dimensions, which were, in any case, lower than 200 nm, slightly increased by increasing the amount of encapsulated NSAID. Indomethacin was also incorporated in the amphiphilic diblock copolymer PVP-poly(d,l-lactide) (PVP-b-PDLLA) by Benahmed et al. [82]. The PVP-b-PDLLA diblock copolymers were prepared by ring-opening polymerization, as also proposed in other works [87,88]. However, the drug dissolution studies were not conducted to understand the release kinetics of the NSAID from the polymeric micelles. Similarly, Fournier et al. [87] encapsulated two anticancer drugs, namely paclitaxel, and docetaxel, in the amphiphilic PVP-b-PDLLA, but no release studies were performed.

In summary, until now, numerous studies were focused on the production of PVP-based microspheres principally for oral drug administration. Nevertheless, the use of polymeric blends, consisting of PVP and other polymers, preferably with a hydrophobic behavior, was investigated in very few papers. However, this route is fascinating for the modulation of drug release, which is increasingly in demand by the pharmaceutical market. In this context, the development of microcapsules can make a significant contribution. Although studied in very few papers, the use of PVP revealed to be very useful for the production of pH-responsive microcapsules for a targeted release. In particular, the use of layer-by-layer capsules seems to be a very promising approach. The production of amphiphilic PVP micelles should also be further investigated. Regarding the technologies, the most recent techniques (such as the supercritical fluids-based ones) allow obtaining a final product free from solvent residues and superior in terms of morphology and particle size distributions. However, it is necessary to take into account the higher costs of these innovative technologies compared to those conventionally employed.

In Table 1, a summary of PVP-based particles proposed to deliver various active compounds is reported. The employed technique, selected carriers, active principles, and morphology and size of produced particles are indicated.

### 2.2. Fibers

The application of fibers has gained considerable interest in several fields, mainly for drug delivery systems, wound dressings, and tissue engineering [4,23,24,89,90,91,92].

In recent decades, the electrospinning process has stood out because of its versatility that allows fabricating fibers from a wide range of materials. Electrospinning is a simple and inexpensive technology for the production of ultrafine fibers with diameters ranging from a few nanometers to several micrometers. Fibers based on different kinds of polymers can be produced using an electrostatically driven jet of polymer solution or polymer melt [22,90,91,92,93,94]. As regards to the pharmaceutical and biomedical fields, drug-loaded fibers can be produced to improve the bioavailability of an active principle, since the electrospun fibers have a high specific surface area and porosity that accelerate the release of the incorporated drug [4,23,24,25,89,95,96]. However, poor resistance to moisture can be one of the main issues related to the use of electrospun fibers in drug delivery [97]. In reverse, the maintenance of a proper moist environment through the electrospun fibers can be exploited to fabricate porous fibrous membranes for wound dressing, also offering good oxygen permeability and protection against microorganisms.

PVP has been used as a polymer matrix for drug-loaded fibers in many papers, by respecting almost all of the aforementioned advantages [4,23,24,25,89,95], but also some critical issues such as the moisture absorption [98].

Rasekh et al. [4] fabricated PVP K90 fibers containing indomethacin (a NSAID) via electrospinning technique for wound care treatment. The fibers were prepared by applying a voltage of 15 ± 2 kV and using a 50/50 *v*/*v* ethanol/methanol mixture. Fixing a PVP concentration in the solution equal to 5% *w*/*v* and 5% *w*/*w* of indomethacin, the effect of the solution flow rate was investigated. It was noted that the mean diameter of PVP/indomethacin fibers produced at a flow rate of 50 mL/min was 2.58 ± 0.30 µm, whereas this size roughly doubled (5.22 ± 0.83 µm) by operating at 100 mL/min. These fibers obtained through the electrospinning process were characterized by diameters appreciably smaller than the existing dressing fibers (about 30 µm). The authors collected the loaded fibers onto three passive dressings, demonstrating their ease of deposition onto any existing normal bandage. In addition, the encapsulation efficiency of indomethacin equal to 75% *w*/*w* was measured. Moreover, the drug was completely dissolved in phosphate-buffered saline solution (PBS) (pH 7.4) in only 45 min, but no comparison with the dissolution profile of unprocessed indomethacin was reported. The topical formulation proposed by Rasekh et al. [4] can be used as an active dressing for a rapid relief system. The fast indomethacin release from PVP fibers can be ascribed to the hydrophilicity and hygroscopicity of PVP; as a result, the polymer–solvent interactions tend to be higher than polymer–polymer forces. Briefly, the solvent was quickly absorbed into the polymer matrix, which swelled, allowing for the rapid release of the drug’s molecules. The authors asserted that the high surface area and the porosity of fibers produced by electrospinning also contributed to speed up the drug dissolution. Similar results were achieved in another study focused on wound healing [23]. Applying a voltage of 10 kV, PVP K90 (10% *w*/*v*) and an antibacterial extract (i.e., Emodin, 0.2% *w*/*v*) were dissolved in ethanol. The attenuated total reflectance Fourier transform infrared analysis suggested the formation of the hydrogen bonds between PVP and emodin, because of the disappearance of the drug characteristic band at 1621 cm^−1^ in the spectrum of medicated fibers. These interactions could improve the stability of the emodin dispersed in the PVP matrix as well as its dissolution. Indeed, a remarkable improvement of the drug dissolution rate was proved by comparing the release profile of pure emodin with the dissolution kinetics of drug released from PVP fibers: embedded emodin was completely released from PVP matrix in 90 min, whereas less than 10% of the pure drug dissolved considering the same time lapse. Furthermore, the in vivo wound healing tests revealed that the emodin-loaded fibers accelerated the wound healing process by about 15 days. In the field of the regenerative medicine, Román-Doval et al. [99] produced scaffolds based on electrospun PVP fibers (average diameter of 135.6 ± 1.4 nm). The electrospun scaffolds have been recently proposed to create artificial skin tissue for the dermal replacement as well as to promote the wound healing process. The results obtained by Román-Doval et al. [99] from the migration assay proved that electrospun PVP fibers favored the cell viability compared to the electrosprayed PVP particles (mean size of 1.0 ± 1.6 µm). Both morphologies were coated with polypyrrole/iodine, which assisted the cell adhesion and accelerated the wound healing, without damaging the cells.

In other studies, electrospun fibers were proposed for oral pharmaceutical formulations [24,25,89]. For example, *Garcinia mangostana L*. extract, which contains various polyphenolic compounds with antioxidant and anticancer properties, was embedded in PVP K90 fibers produced by electrospinning method [24]. Both unloaded polymer fibers and fibers loaded with the active principle were produced using a voltage of 15 kV, ethanol as solvent, 10% *w*/*w* of PVP, and 2% *w*/*w* of extract in the electrospun solution. The mean diameter of PVP fibers was lower than 480 nm, whereas the addition of the active principle increased the average diameter at 690 nm. It was demonstrated that 80% of *Garcinia mangostana L.* extract was released in PBS (pH 6.8) in 30 min, which is an advantage for the application of loaded fibers as drug delivery systems. However, there is a criticality linked to the solution flow rate employed equal to 1 mL/h; indeed, the electrospinning process took about 8–10 h to process only a 10 mL solution. Therefore, the processing time represents a great limit considering a large-scale production. This issue emerged from most of the works focusing on the electrospinning of drug-loaded PVP fibers, in which the solution flow rate ranged between 0.2 and 2 mL/h [23,25,89,96,100,101].

Yu et al. [25] also prepared ibuprofen-loaded PVP fibers by electrospinning to produce oral fast-dissolving drug delivery systems. The medicated fibers were prepared by using a voltage of 15 kV, a PVP K30 concentration in ethanol equal to 40% *w*/*w* and different amounts of ibuprofen in the solution (0, 7.5 and 15% *w*/*w*), which was delivered with a flow rate of 2 mL/h. The dissolving properties of the composite fibers were studied by placing the samples in water and monitoring the disappearance of the medicated fibers visibly. Indeed, after 10 s, fibers dissolved thanks to a polymer-controlled mechanism. However, no release kinetics related to in vitro dissolution tests was reported to verify how ibuprofen moves away from PVP fibers.

To tune the physical and mechanical properties of electrospun fibers, PVP has been coupled with other polymers in several studies [95,96,100,101,102,103,104,105,106]. Zein, a protein contained in corn, has been commonly studied as a polymer to be combined with PVP [96,102,103]. Due to its hydrophobicity and good elasticity, electrospun zein fibers have recently attracted attention for biomedical applications (e.g., scaffolds) and novel drug delivery systems [107,108,109,110,111].

Jiang et al. [96] aimed to produce core–sheath fibers by coaxial electrospinning to provide a biphasic drug release profiles, i.e., to release a drug at two different rates. Indeed, in the case of some pathologies, an immediate drug release followed by a sustained release may be the best option. In essence, the initial fast release favors the relief of the symptoms, then a constant release of the remaining dose can avoid repeated administration. Jiang et al. selected PVP K60 as polymeric sheath and zein as core matrix, whereas ketoprofen (the model drug) was charged at 2% *w*/*v* both in core and sheath fluid. The core part had a mean diameter of 730 ± 190 nm, while the sheath had a thickness of about 90 nm, which were measured from SEM and and Transmission Electron Microscopy (TEM) images The dissolution profile of the core–shell structure was compared with fibers made up of PVP alone, which promotes a rapid release of ketoprofen, and with fibers made up of only zein, characterized by a drug-prolonged release without burst-like effect [77]. The core–shell fibers immediately released about 42% of ketoprofen, followed by a sustained release of the remaining drug up to 12 h. In another paper [102], again, ketoprofen was loaded in PVP K60/zein fibers, but, differently, the two polymers were blended in the same ethanol aqueous solutions, to prepare a well-distributed binary polymer. Composite fibers were prepared at different PVP/zein ratios, from 16/84 to 40/60 *w*/*w*. In vitro dissolution tests clearly demonstrated the effect of PVP amount; indeed, the dissolution rate of ketoprofen enhanced by increasing the PVP quantity in the composite fibers, as reported in other works [102,105].

In other studies, the hydrophilic PVP was combined with polymers characterized by a hydrophobic behavior. For example, PVP/polycaprolactone (PCL) fibers were fabricated for wound dressings [101], because PCL offers strength to the matrix, in addition to having good tissue compatibility and excellent electrospinnability. The bactericidal properties were imparted to PVP/PCL fibers by incorporating an herbal extract of *Tecomella undulata*, which was tested against common pathogens such as *Pseudomonas aeruginosa*, *Staphylococcus aureus,* and *Escherichia coli*. To produce composite fibers, the same weighted quantities of PCL and PVP were dissolved in the solution (chloroform/methanol 4/1), by adding 7.5% of the antimicrobial agent with respect to the weight of the polymers. This mixture was electrospun, keeping the voltage at 12 kV and the solution flow rate at 0.75 mL/h. The release of the antibacterial agent was prolonged up to 24 h. In other studies, the electrospinning of PVP/PCL blends was applied for tissue engineering and regenerative medicine [13,112,113].

Samprasit et al. [106] prepared PVP/cyclodextrins (CDs) fibers loaded with meloxicam, a NSAID, to be applied as oral drug delivery systems. The main purpose was to mask the drug taste, in addition to improving its dissolution rate. Therefore, menthol and aspartame were added to the electrospun solution as flavoring and sweetening agents, respectively. It is well-known that CDs allow masking unpleasant tastes and increasing the dissolution rate of poorly water-soluble compounds, in addition to being processable by electrospinning technique [114,115,116]. In particular, in the study of Samprasit et al., β-CD or H-β-CD (hydroxypropyl-β-CD) were blended with PVP, leading to an improvement in terms of morphology and stability of PVP-based fibers [106]. Moreover, CDs offered protection from moisture, observing a decrease in the hygroscopic effect of PVP. Rapid disintegration and fast release of meloxicam were observed in the case of composite fibers. The drug contained in the fibers took about 60 min for the complete dissolution, resulting in a faster dissolution with respect to the pure drug and the commercial formulation. The disintegration time and taste masking were also evaluated through in vivo tests, thanks to healthy human volunteers.

In summary, PVP-based fibers appear to be a truly versatile morphology, essentially obtained via the electrospinning process. The electrospun PVP-based fibers revealed to be particularly promising as innovation in regenerative medicine, especially for the tissue regeneration process. Unfortunately, it has emerged that this technique has strong limitations from an industrial point of view; that is the long process times due to the low flow rates of the solution used to obtain fibers with the proper characteristics. Reducing the working time while maintaining an optimal fiber morphology represents the main challenge. Instead, from a pharmaceutical point of view, the possibility of having a biphasic release of one or more drugs is attractive for various applications.

**Figure 2:**
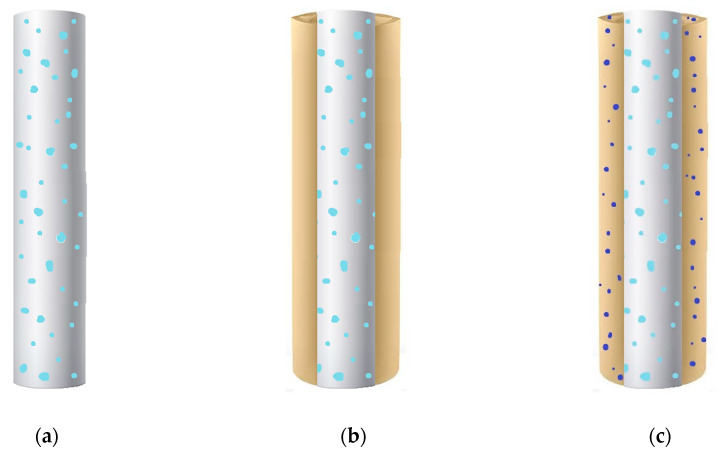
Possible PVP-based fibers: (**a**) active principle dispersed into the polymeric matrix; (**b**) drug dispersed into the core of a core–shell structure; and (**c**) drug dispersed both into the core and the shell of a core–shell structure.

A sketch of different drug-loaded fibers that can be obtained by using PVP is reported in Figure 2. The active principle can be dispersed into the polymeric matrix, consisting of PVP or blends, as shown in Figure 2a. Instead, in the case of core–shell fibers, the drug can be dispersed only into the core material (Figure 2b) or both into the core and the shell (Figure 2c); in the latter option, the same active principle or even a different one can be loaded in the sheath. The main results obtained in the papers focused on the production of PVP-based fibers are summarized in Table 2; the employed technique, the polymeric carriers, and the incorporated active compounds are specified.

### 2.3. Hydrogels

Hydrogels are polymeric networks that can absorb huge amounts of water thanks to the hydrophilic groups, causing the swelling phenomenon. However, the structure of the hydrogels is preserved by the crosslinking between the polymeric chains [117]. Hydrogels represent a useful matrix for the entrapment of drugs, offering unique advantages such as biocompatibility, low toxicity, and flexibility in controlling the diffusion properties of drugs. However, there are also some drawbacks, such as the possibility of a too rapid drug release from large porous hydrogels or during the hydrogel swelling in an aqueous environment. Besides, the residues of unreacted small-molecule crosslinkers in the case of hydrogel production by the small-molecule crosslinking method may be toxic, and the dispersion of hydrophobic drugs within the hydrogels may be non-homogeneous [118]. Hydrogels were proposed in pharmaceutical and biomedical fields as oral drug delivery systems, for transdermal and topical applications (e.g., wound dressings), nasal and ocular route (e.g., particular contact lenses), and implants [118,119,120,121,122]. Among the first, Rosiak et al. [123] found a methodology to produce hydrogel-based dressings by the polymerization and crosslinking. These dressings were produced with mixtures of natural and synthetic polymers, including PVP. Up to now, PVP hydrogels were produced by using different methods [124,125,126,127]. However, only a few works were focused on the production of PVP-based hydrogels for drug delivery [5,26,27,28,29]. For example, PVP K30/pectin hydrogels were prepared by the traditional solution casting technique [26], and then the salicylic acid (the model drug) was incorporated by diffusion method; i.e., the hydrogel is immersed in a solution containing the drug. The formation of hydrogen bonds between the two polymers was observed through FT-IR (Fourier transform infrared) analysis. Moreover, an increase in the hydrogel strength was noted by increasing the PVP amount. Swelling properties and drug release were studied at different pH values (1.4–7.4). A slightly faster drug release was observed at the higher pH value. Indeed, an initial burst was noted up to 15 min, when 65% and 55% of the drug were released at pH 1.4 and 7.4, respectively. This behavior was correlated to the major swelling in the alkaline environment with respect to the acid one. Moreover, the maximum swelling was reached at the higher PVP/pectin ratio. It is possible to state that, although to a small extent, a sort of pH dependence was obtained in the case of PVP/pectin hydrogels produced by Mishra et al. [26].

Recently, hydrogels responding to external stimuli, including heat and pH, attracted the attention of many researchers [28,29]. In the case of pH-responsive hydrogels, the drug release is triggered by pH changes, as occurred in the different parts of the human body. In this context, Ajji et al. [28] proposed a PVP hydrogel grafted with crotonic acid, aiming to obtain new pH-responsive systems. Choosing ketoprofen as the model drug, the release behavior of drug-loaded hydrogel was studied in two different release media; i.e., at pH 1 and 7.2. Various concentrations of crotonic acid were added to the PVP solution, proving an improvement of the swelling property of the PVP-based hydrogels by increasing the amount of crotonic acid. As for the dissolution tests, the percentage of the released drug was limited in the acid environment (about 25% in 3.5 h) with respect to the extended release observed in the buffer solution at intestinal pH equal to 7.2 (the plateau value reached after about 19 h). The authors correlated the pH dependence of the release with the presence of hydrogen bonds in the acid medium due to the protonated hydroxyl groups. In contrast, the hydrogel network expanded in the alkaline medium because of a decrease of the hydrogen bonds and, therefore, the drug can be freely released. The hydrogel swelling was investigated at different pH values since it plays a key role in the release kinetics of the drug from the hydrogel. It was noted that the swelling capability significantly increased by increasing the pH value. From a pharmaceutical point of view, the irritation of the stomach mucosa caused by ketoprofen is minimized thanks to the pH-responsive hydrogel.

Similarly, pH-sensitive PVP K30/chitosan hydrogels were prepared for the release of antibiotics in the gastric environment [29]. The aim was to eradicate *Helicobacter pylori* efficiently, which is one of the main causes of peptic ulcers when these bacteria colonize the gastric mucosa. Amoxicillin was selected as a model drug, being effective in treating *H. pylori*. Chitosan, a cationic polysaccharide, was blended with PVP since cationic swellable-hydrogels are effective in the stomach-targeted release [128]. Comparing the hydrogels prepared by the freeze-drying and air-drying methods, the dependence of swelling from the pH value was higher in the first case. For both types of hydrogels, it was noted that acid media (pH = 1 and 2) caused a more significant swelling with respect to neutral and slightly-basic media (pH = 7 and 9.2, respectively). This behavior was explained through FT-IR analyses, which highlighted the protonation of a primary amino group on chitosan in the case of hydrogels swollen in the acid media, proving the polymer chain relaxation. Amoxicillin released from freeze-dried hydrogels exhibited the best dissolution kinetics in acid environments (pH = 1.0, 2.0, and 3.0), in agreement with the literature [129]. Indeed, at a pH value equal to 1.0, 73.2% and 31.7% of the incorporated antibiotic was released in about 2 h from freeze-dried and air-dried hydrogels, respectively. This outcome can be attributed to high porosity and, consequently, to a high specific surface area of freeze-dried hydrogels that lead to better matrix–solvent interaction, resulting in rapid swelling and drug release.

Fogaça and Catalani [5] proposed a new approach to develop PVP hydrogels, based on PVP K90 nanofibers produced via electrospinning and then crosslinked by UVC radiation (wavelength = 254 nm) and Fenton reaction. In this way, very porous structures in which bovine serum albumin (BSA) or collagenase were incorporated were produced. These hydrogels showed good swelling properties, slightly higher in the case of Fenton-crosslinking than in UV crosslinking. The fast release of BSA from PVP fibers-based hydrogels was compared to the slow release of BSA from PVP casting hydrogels. Results were explained through the drug release modeling: the BSA release from fibrous hydrogels is only governed by Fickian diffusion, whereas the BSA release from cast hydrogels is governed by the polymer chain relaxation (Case II transport). The dissolution tests were also performed on collagenase-loaded hydrogels produced with electrospun fibers. Most of the collagenase was release in 10 h; the remaining part reached a plateau value in 48 h. The authors proposed collagenase-loaded PVP hydrogels prepared by electrospinning for wounds and burns treatment. Some advances have been made in regenerative medicine and tissue engineering using PVP-based hydrogels [15,130,131,132]. In this context, Goetten de Lima et al. [15] prepared a novel hydrogels-based dressing using PVP, polyethylene glycol (PEG), agar, and carboxymethyl cellulose (CMC). Silver ions were dispersed in the polymeric network, and the gamma irradiation was employed to obtain the hydrogel loaded with silver nanoparticles. The incorporated nanoparticles enhanced the antimicrobial properties of dressings and promoted a faster wound healing process compared to the untreated wounds. However, in this study, the loading of an active principle that may further accelerate the wound healing was not attempted. Instead, the work of Shah et al. [131] proved that hydrogels responding to external stimuli, like pH and temperature, are also interesting in the biomedical applications. In particular, biomineralized (CaCO_3_) PVP/CMC hydrogels were prepared for bone tissue engineering. The PVP/CMC hydrogel was prepared via solution casting. Then, the liquid diffusion technique was applied to achieve a biomineralized hydrogel; i.e., the PVP/CMC hydrogel was immersed in an ionic solution consisting of Na_2_CO_3_ and CaCl_2_. This last step allowed the organization of calcite (CaCO_3_) crystals inside the hydrogel network. The swelling response of biomineralized hydrogels was studied in aqueous solution in the ranges of temperature and pH equal to 10–40 °C and 4–9, respectively. It was observed that the maximum swelling was reached in the range of temperatures 30–40 °C and at pH equal to 7. Moreover, the physical changes of hydrogels were also studied by immersing them in different simulated biological solutions containing glucose, urea, or physiological fluid, at 37 °C and pH equal to 7.5. The hydrogel swelling was found to be higher in the urea solution, followed by the physiological solution and, at last, the glucose one. The authors indicated that the swelling behaviors of hydrogels in the simulated biological solutions and their non-reformative structure validated the possible use of the biomineralized PVP/CMC hydrogels for bone tissue engineering and regenerative medicine.

As discussed above, the literature available on PVP-based hydrogels is still limited. Until now, this type of system has been proposed mainly for the oral drug administration as pH-responsive hydrogels; its use has also been suggested for topical formulations and in tissue engineering. However, PVP-based hydrogels could be truly innovative for pharmaceutical applications when other morphologies are not appropriate, such as in the case of medicated ocular systems.

**Figure 3:**
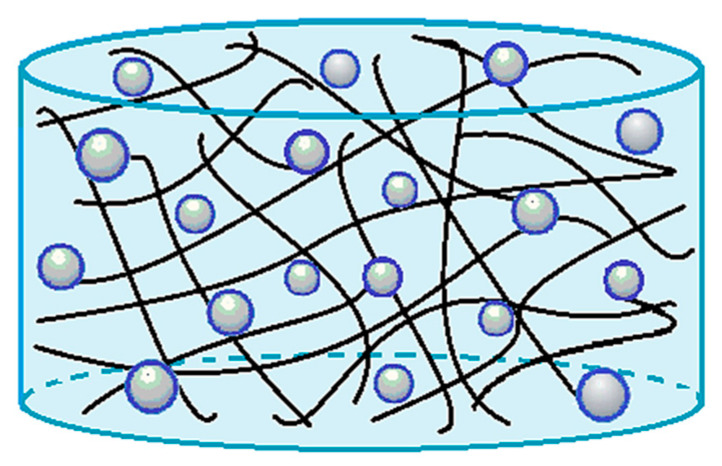
A sketch of PVP-based hydrogels.

In Figure 3, a sketch of drug-loaded hydrogels is reported, showing the molecule of active principle dispersed in the network (PVP or polymeric blends).

In Table 3, a summary of the main results reached up to now by producing PVP-based hydrogels is reported, indicating the employed technique, the selected carriers, and active compounds for each study.

### 2.4. Tablets

**Figure 4:**
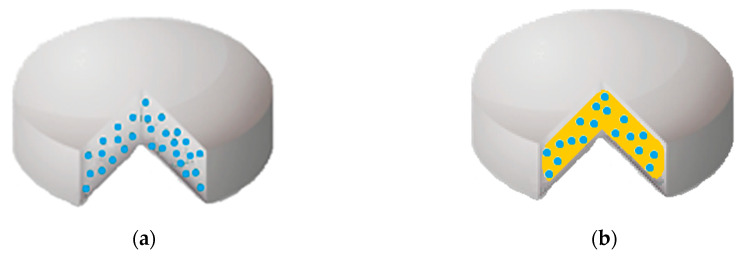
Possible PVP-based tablets: (**a**) monolithic matrix systems; and (**b**) osmotic systems or core–coating structure.

Polymeric tablets are matrices with great potential for oral controlled drug delivery, in addition to having rather low manufacturing cost [133]. There are two different kinds of tablets, namely monolithic matrix systems (Figure 4a) and osmotic systems (Figure 4b). In the former (Figure 4a), the drug is dispersed in the polymeric matrix, which can be predominantly hydrophobic or hydrophilic [133]. Instead, in the latter, the osmotic systems consist of a core tablet surrounded by a semipermeable coating, sketched with a different color in Figure 4b. The inner core usually contains the active principle, thus called the active layer, and the external barrier layer contains the osmotic agent. The release of the active compound from the polymer matrix will be strongly influenced by the type of tablet. Indeed, the drug release from the oral tablets can occur through different mechanisms, depending on whether they are erodible matrix (size reduction) or swellable matrix (increase in tablet volume and drug diffusion) [133].

Drug-loaded tablets are often obtained by compressing polymer–drug solid dispersions produced through various techniques [30,134,135]. The direct compression of the polymer–drug solid dispersions turned out to be a simple and effective method to produce pharmaceutical tablets. Wlodarski et al. [30] applied two different processes, namely spray drying and ball milling, to prepare amorphous PVP/vinyl acetate (VAc) solid dispersions; then, tablets were produced by the direct compression of the composite powders. Immediate-release tablets containing different quantities of tadalafil (a model drug), in the range 2.5–20 mg, were prepared by fixing a polymer/drug ratio 1/1 *w*/*w*. Drug loaded-tablets produced from both the different solid dispersions led to a significant increase of the tadalafil dissolution rate, compared to tablets containing drug in the crystalline state and to commercial formulations. This improvement resulted in being more marked the lower the drug content, probably due to the formation of a gelling network on the tablet surface in the dissolution media at high drug content. Moreover, at low drug content (2.5 mg), the spray-dried solid dispersions exhibited better release profiles with respect to the ball-milled one, whereas the opposite occurred at high drug content (20 mg). The dissolution rates of tadalafil from different dosage formulations were also compared, resulting as follows: powders > tablets > capsules. According to the authors, this difference might be explained through the presence of excipients (e.g., disintegrants and fillers) in the formulations with solid dispersions, avoiding the aggregation of particles. These aggregates were instead formed in the case of capsules, leading to a worsening in dissolution.

Nowadays, the solid dispersions forming the tablets can also be obtained with alternative technologies, e.g., the solvent-free impregnation with supercritical CO_2_. Indeed, solid dispersions of piroxicam, a poorly-water soluble NSAID, with PVP were produced via supercritical impregnation, working at 300 bar and 100 °C [135]. Different molecular weights PVP (PVP K15, K30, and K90) were tested to investigate the role of the molecular weight of the polymer on the formulation preparation and the drug dissolution kinetics. After the impregnation tests, PVP/piroxicam samples were compressed into cylindrical tablets. The release profiles of the drug from the tablets showed a significant increase in the dissolution rate with the PVP K15/piroxicam tablets, containing a drug amount lower than 13% (*w*/*w*). This positive effect of PVP on the drug release decreased as the molecular weight of the polymer increased until no changes or even a reduction in the dissolution rate were observed by using PVP K90, because of the formation of a superficial gelified-layer when in contact with the dissolution medium. This result was explained through the possible formation of strong molecular interactions between the active principle and the long-chain polymer under supercritical conditions.

PVP-based tablets characterized by an inner core and an external coating (or barrier layer) have also been proposed [31,32,136,137]. Rabiu Yakubu and coauthors [136] produced PVP-based osmotic tablets to reach a 24 h-controlled release of ketoprofen (model NSAID). The composite granules prepared by wet granulation were compressed to form the tablet core, composed of drug and PVP K30, dextrose, and MCC (microcrystalline cellulose) as polymeric carriers. Different polymer–drug ratios were investigated for the tablet core; the influence of PVP content was the main parameter investigated. Firstly, dip-coating, which consists of immersing the tablet in a solution of the coating materials, was employed to study the effect of concentration of PVP as an osmotic and suspending agent. Then, spray-coating was employed for the final coating formulation, consisted of PVP K30, cellulose acetate, and triethyl citrate (TEC). An increase in the drug release rate was observed as the PVP concentration into the tablet core increased, from a PVP/ketoprofen ratio of 9/1 to 5/1 *w*/*w*. This trend was due to an increase in the generation of osmotic pressure by increasing the PVP content. Moreover, the results indicate that these PVP amounts were suitable to suspend the drug molecules in the tablets. On the contrary, the release profiles of ketoprofen from tablets containing a PVP/drug ratio of 1/1 and 3/1 *w*/*w* exhibited the slowest release, probably because the PVP content was not sufficient for a good suspension of the drug molecules into the tablets. It was proved that PVP also offers a binding effect: indeed, in the case of the tablet cores without PVP, the coatings broke down in about 10 min before the start of the analysis sampling. This outcome could be explained considering that the binding effect of PVP probably avoided the rapid dissolution of dextrose into the release medium and the consequent sudden increase in the osmotic pressure, which caused the premature breakage of the coating. Furthermore, the percentage of released ketoprofen enhanced by increasing the PVP concentration in the coating because of the increased pore formation from which the drug can be released. The tablets coated with PVP were also compared with tablets prepared by using other polymers as pore-formers into the coating, namely polyethylene glycol (PEG) and lactose. It was observed that the release kinetics from tablets coated with PVP or PEG were similar, reaching about 95% in 24 h; these release profiles were superior to that obtained with a lactose-based coating (about 24% in 24 h) or without any polymeric pore-former into the coating. PVP and PEG dissolved in the dissolution medium with the consequent formation of orifices into the coating, allowing a faster drug diffusion. The authors also studied the influence of the molecular weight of PVP on the drug dissolution, comparing different molecular weights PVP (K25, K30, and K90). After a period of 24 h, about 95%, 65%, and 55% of ketoprofen were released from tablets containing PVP K30, PVP K90, and K25, respectively. In general, for a hydrophilic polymer such as PVP, the viscosity increases while the solubility decreases by increasing the polymer molecular weight. The results of the dissolution tests proved that not only the PVP solubility has a positive influence on the osmotic pressure and drug dissolution, but also the viscosity strongly affects the release of the poorly water-soluble drug. A too low viscosity does not allow the desirable suspension of the drug inside the polymer matrix, thus explaining the higher release rate produced by PVP K90 compared to PVP K25. PVP K30 revealed to be a good compromise in terms of solubility and viscosity, therefore more suitable to produce osmotic tablets for a controlled drug release within 24 h. In another work [32], the method of solvent evaporation followed by compression was used to form both the tablet core and the coating, which consisted of PVP/Felodipine and PVP/hydroxypropyl methylcellulose (HPMC), respectively. It was noted that the high water solubility of PVP limited its mucoadhesion; however, the use of PVP/HPMC blends improved the mucoadhesive properties of both the polymers. In particular, the maximum adhesive force was reached with coatings PVP/HPMC at ratios between 30/70 and 50/50 *w*/*w*. Moreover, the authors asserted that PVP imparted good flexibility to the coating, avoiding its collapse. From the in vitro dissolution tests, it was observed that the delay time that characterized the release profiles reduced by increasing the PVP content from 30% to 90% by weight.

A novel tool for the manufacture of PVP-based tablets is the 3D-printing [138,139]. For the first time, Okwuosa and coauthors [138] fabricated 3D printed tablets by using PVP K30 as the polymeric carrier, to achieve a rapid release of dipyridamole or theophylline (selected as model drugs). It was demonstrated that drug-loaded tablets could be fabricated at a temperature as low as 110 °C, thus overcoming the main drawbacks in 3D-printing of pharmaceutical systems, mainly the required high temperature (>200 °C) and, consequently, the low drug loadings [140,141,142,143]. Triethyl citrate was used as the plasticizer. All the 3D-printed PVP tablets revealed to be mechanically strong, showing no friability, in addition to an acceptable in-batch variability. By immersing the tablets in aqueous solutions, the disintegration time was found to be less than 15 min for all the 3D-printed samples. Both the studied drugs were quickly released from PVP tablets in about 40 min; however, these profiles were not compared with the dissolution profiles of the unprocessed drugs to highlight the effect of PVP. As reported in Kempin et al. [139], other parameters influence the release of the active principle from 3D printed PVP tablets; for example, it was noted that the drug dissolution rate from PVP tablets increased by decreasing the infill rate during the printing, due to an increase in tablet porosity.

In summary, the production of oral tablets is quite consolidated. The release of drugs can be tuned using core–coating tablets or blends containing PVP and other hydrophobic polymers. The use of polymeric blends also allows improving the tablet features, such as mechanical and disintegration properties. Most recently applied, the 3D printing of pharmaceutical formulations can offer flexibility in the production and reduced process times.

For each work focused on PVP-based tablets, the main results are summarized in Table 4, with the indication of the technique and the materials employed.

### 2.5. Films

In recent years, thin films gained increasing interest as emerging support for drug delivery. Films are excellent polymeric matrices for the targeted drug release, which is often not possible with liquid formulations, tablets, or traditional oral administration [145,146]. Thin films are accepted by patients due to their flexibility as well as the reduced thickness and encumbrance. These non-invasive formulations offer numerous advantages, such as easy handling during manufacture and transportation and moderate costs in the development of formulations. However, an ideal film has to possess some peculiar properties, such as sufficient drug loading capacity, formulation stability, and relatively long residence time in the site of administration. Besides, it has to be non-toxic and biocompatible.

PVP possesses excellent film-forming property [6,33,34,35,36,98,147,148,149,150]. Up to now, PVP-based films were essentially produced via solution casting, followed by solvent evaporation. Using this method, del Consuelo et al. [6] prepared bioadhesive film for the buccal administration of fentanyl (an analgesic). Firstly, monolayer-loaded films were produced with PVP K30 or PVP K90; these films consisted of 1.28 mg of the active principle and 60 mg of polymer. Then, bilayer films were prepared by covering the drug-loaded PVP K90 films on one side with an insoluble layer of Eudragit RS, which acted as an impermeable backing layer. Therefore, the active principle content with respect to the polymer’s amount was low in the final films. Moreover, PVP films were brittle due to the absence of a plasticizer, especially in the case of PVP K30. Indeed, it was observed that the film brittleness increased by decreasing the PVP molecular weight. However, the contact with the buccal tissue made the PVP film more flexible thanks to the water uptake. In vitro drug dissolution tests showed that the release of fentanyl was influenced by the molecular weight of PVP since the drug was released more quickly by the PVP K30 (in about 20 min) than by the PVP K90 films (in about 60 min). All the films exhibited good mucoadhesive properties since they were difficult to remove after an 8 h application. Moreover, it was proved that the addition of the backing layer of Eudragit RS did not affect the drug release kinetics.

There are other few works focused on the use of films containing PVP for buccal drug delivery [33,147]. For example, ibuprofen was loaded in films constituted by PVP K90 as the film-forming polymer, carboxymethylcellulose sodium salt (NaCMC) as the mucoadhesive polymer, and propylene glycol as the plasticizer [33]. In this study, PVP-based films resulted in being more suitable for buccal administration with respect to films produced with different insoluble Eudragits, thanks to rapid hydration of the former after the application in the oral cavity and homogeneous miscibility of the mucoadhesive polymer in the PVP matrix. Besides, PVP/NaCMC films revealed to be superior than brittle PVP/HPMC films due to the higher water uptake, which leads to a better diffusion of drug from PVP/NaCMC films towards tissues. It is worth noting that these buccal delivery systems allow to avoid the gastrointestinal irritation caused by oral formulations.

Nevertheless, PVP-based films are most often applied to develop transdermal patches [34,35,36,98,148,149,150]. For this purpose, PVP was blended with a wide variety of polymers, mainly ethyl cellulose (EC) [34,35,36,148]. Indeed, Sadashivaiah et al. [35] fabricated transdermal patches loaded with haloperidol, an antipsychotic drug suitable for schizophrenia, by using PVP K30 and EC as film-forming polymers. To produce films at different PVP/EC ratios (from 1/4 to 4/1 *w*/*w*), dibutyl phthalate (30% with respect to the polymers’ weight), and hyaluronidase (4% *w*/*w*) were added as plasticizer and permeation enhancer, respectively. The drug release rate increased by increasing the PVP concentration in the films, since the addition of the hydrophilic polymer to the insoluble EC led to the formation of pores in the film in contact with the dissolution medium, so reducing the mean diffusion path length of the drug molecules. The same trend to improve drug dissolution with increasing PVP content in films was also observed in other studies [98,148,149,150]. The higher dissolution rate of haloperidol was also attributed to the PVP anti-nucleating effect, as also indicated in another work [149]. It is, therefore, possible to deduce that the drug contained in the matrix was in an amorphous state since PVP retards or inhibits the drug crystallization. The presence of PVP resulted in a low moisture uptake, which, according to the authors, improved the stability of the film by making it less fragile. The formulation PVP/EC 2/1 showed one of the best release kinetics for a sustained release lasting one day.

PVP was also blended with polymers of different nature; for example, it was paired with polymerized rosin, a solid resin naturally obtained from pine trees, poorly evaluated for transdermal delivery despite its good film-forming property [98]. Diltiazem hydrochloride, a calcium channel blocker selected as a model drug for the treatment of arrhythmia and hypertension, was loaded in PVP/rosin films (4/6, 3/7, and 2/8 *w*/*w*) [98]. In all formulations, 30% of dibutyl phthalate was used as the plasticizer. It was observed that the moisture content and the water absorption capacity of patches increased by increasing the concentration of the hydrophilic PVP. However, a high absorption capacity of films leads to a higher risk of microbial contamination of patches. This drawback about the increase of moisture content in the PVP-based films by increasing the PVP amount was also highlighted in other works [36,150]. Moreover, Satturwar et al. [98] asserted that the tensile strength increased by increasing the PVP amount, enhancing the risk of film cracking. Nevertheless, no cracking was observed in the obtained films, thanks to the presence of the plasticizer. It was also noted that, by increasing the PVP concentration in the films, the dissolution rate enhanced, and a higher initial burst release was also observed. Again, the addition of the water-soluble PVP to the insoluble films caused the formation of pores in the structure during the dissolution studies; this led to an enhancement in the drug release rate, as explained in another work of Sadashivaiah et al. [35]. The in vitro drug permeation tests proved that the permeation of the drug across the skin improved by increasing the PVP concentration in the formulation. Other authors demonstrated the rising improvement of the skin permeation as the PVP content in the films increased [148,149].

In summary, PVP exhibits excellent film-forming properties. Thin PVP-based films are suitable for topical or transdermal applications, in addition to buccal drug delivery. However, the high absorption of humidity due to the strong hydrophilicity and hygroscopicity of the PVP can be a serious problem. Indeed, a high water uptake can lead to microbial contamination, making medicated patches practically unusable or even harmful. In this context, the study of new polymeric blends is required, thus also improving the mechanical properties of the films.

**Figure 5:**
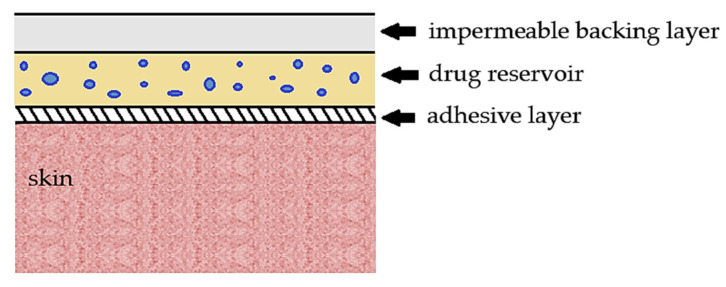
A sketch of a possible medicated patch.

In Figure 5, a sketch of a medicated patch obtained using PVP is reported. It can consist of an adhesive layer, a drug reservoir, i.e., the drug dispersed in a polymeric film (PVP or blends), and a backing layer (generally based on hydrophobic polymers), which may be present or absent.

The main results obtained until now in the production of films by using PVP are reported in Table 5. The employed technique, the selected carriers, and active compounds are also indicated.

## 3. Conclusions and Perspectives

This review focuses on the rule of PVP in the delivery of active principles. PVP offers numerous benefits in the pharmaceutical and biomedical fields, as a stabilizer, protecting agent, crystallization inhibitor, and dissolution enhancer, but also some critical issues must be controlled, mainly the moisture adsorption. PVP is used both alone and in blends with other polymers and for composite systems characterized by a core–shell structure or a coating.

Different morphologies and formulations were obtained by different technologies. Each morphology was found to be more or less suitable for a specific type of drug administration. For example, PVP tablets were applied for oral formulations, as well as microparticles and nanoparticles. The reduction in particle size combined with the use of PVP as a hydrophilic matrix or shell allowed improving the dissolution of many poorly-water soluble active compounds significantly. As concerned PVP-based tablets, both monolithic matrix systems and osmotic systems were produced to achieve different release rates.

Other morphologies are more versatile, such as the PVP-based fibers proposed for both topical formulations or tissue engineering and oral route. They allow us to reach a fast release (mainly, with PVP only), to tune the release (e.g., with PVP/hydrophobic polymers blends), or even to obtain a biphasic drug release (i.e., both immediate and prolonged release, generally with core–shell fibers). Hydrogels were also proposed for different formulations, such as oral, transdermal, or topical routes. Besides, pH-responsive hydrogels were obtained to release the drug in a specific part of the body, thanks to the swelling properties of PVP-based hydrogels at different pH values. PVP-based thin films were also developed for various applications, including buccal, sublingual, and skin routes. This non-invasive system is generally well-accepted by patients and guarantees a targeted drug release.

Basically, up to now, low-medium molecular weight PVP was mainly used for the attainment of microparticles (from PVP K10 to PVP K30). In contrast, medium-high molecular weight PVP was employed for the other formulations (from PVP K30 to PVP K90).

The literature clearly shows that the production of PVP-based particles was widely investigated by using several techniques. However, PVP was mainly employed alone, whereas blends of PVP with other polymers were proposed to produce drug-loaded particles only in a few papers. The literature involving the production of PVP-based fibers and tablets is quite satisfactory. In particular, the fibers are a particularly interesting morphology, given to their versatility. However, studies concerning PVP-based fibers proved that they are particularly subject to the moisture absorption; therefore, the development of blends with other polymers is advisable to increase the moisture resistance of fibers. Few studies were instead focused on hydrogels production using PVP as a carrier. However, they were found to be very interesting to produce of pH-sensitive systems. Moreover, the use of PVP-based hydrogels has not yet been proposed for ocular drug delivery, although hydrogels are one of the promising systems for this purpose. Regenerative medicine also emerged as an innovative area of applications of fibers and hydrogels based on PVP. The production of thin films using PVP as a carrier is also an innovative field for various pharmaceutical applications. These systems allow a controlled drug release, reducing the side effects associated with the oral administration. Therefore, the use of blends of PVP and other hydrophobic polymers can be further investigated to tune the release of the active compounds from films.

## Figures and Tables

**Table 1:** PVP-based particles. HP-β-CD, hydroxypropyl-β-cyclodextrin; MCs, microcapsules; MPs, microparticles; m.s., mean size; NPs, nanoparticles; PMMA, poly(methyl methacrylate); MPR, m-methylphenol-formaldehyde resin; PAA, poly(acrylic acid); PAH, poly(allylamine hydrochloride); PMA, poly(methacrylic acid); SMPs, submicroparticles; PMs, polymeric micelles; PDLLA, poly(d,l-lactide).

Technique	Polymeric Carrier	Active Compound	Results	Reference
Spray drying	PVP	Andrographolide	MPs with m.s. in the range 2.8–3.6 µm	[2]
	PVP/meglumine	Celecobix	slightly rough MPs with m.s. in the range 3–5 µm	[18]
	PVP	Curcuma Extract	rough spherical MPs	[3]
	PVP	Curcumin	collapsed MPs	[38]
	PVP	Naproxen	no SEM images reported	[60]
	PVP	Probucol	collapsed MPs with m.s. in the range 7.4–9.0 µm	[39]
	PVP/HP-β-CD	meloxicam	collapsed and slightly coalescent MPs (m.s. 2.52 µm)	[65]
Freeze-drying + ultrasound assisted spray-congealing	PVP/stearic acid	Indomethacin	crystals for freeze-dried PVP/drug; rough MCs PVP/stearic acid/drug	[63]
Coacervation	PVP or PVP/polystyrene	2-propylpyridine, 4-nitroanisole, acridine, Sudan 2 and Sudan 3	no SEM images reported	[19]
	PVP	4-nitroanisole and methylene blue	pH responsive MCs	[40]
Dispersion Polymerization	PVP/PMMA	Cefadroxil and indomethacin	spherical drug-loaded PMMA microspheres coated with PVP	[62]
Layer-by-layer method	PVP/MPR with and without a PAA/PAH bilayer	-	hollow MCs (m.s. of PVP/MPR MCs about 440 nm)	[70]
Layer-by-layer method	PVP/PMA	Rifampicin	eight-layered MCs (size about 4 µm)	[71]
SAA process	PVP	Curcumin	collapsed SMPs and MPs with m.s. in the range 0.54–0.76	[46]
	PVP	Luteolin	collapsed SMPs with m.s. in the range 0.22–0.33 μm	[47]
	PVP	Propolis	SMPs with m.s. in the range 0.23–0.50 µm	[48]
	PVP	β-carotene	MPs and SMPs with m.s. in the range 0.28–0.84 μm	[49]
spray drying or SAS process	PVP	Piroxicam	SAS MPs (0.1–5.0 µm); spray dried MPs (0.3–8.0 µm)	[20]
SAS process	PVP	Cefuroxime axetil	both coalescent and well-separated MPs (m.s. in the range 1.88–3.97 µm)	[50]
	PVP	Curcumin	NPs and SMPs with m.s. in the range 0.03–0.34 µm	[41]
	PVP	Dexamethasone, prednisolone and budesonide	Dexamethasone MPs (m.s. 1.82–2.51 µm), prednisolone MPs (m.s. 1.96–3.03 µm) and budesonide MPs (m.s. 3.06–3.58 µm)	[21]
	PVP	Nimesulide	aggregates or MPs (m.s. 1.67–4.04 µm)	[51]
	PVP	α-tocopherol and menadione	α-tocopherol MPs (m.s. 1.80–4.08 µm) and menadione MPs (m.s. 2.64–5.09 µm)	[42]
	PVP	Folic Acid	SMPs and MPs with m.s. in the range 0.30–3.80 µm	[52]
	PVP	β-carotene	NPs (0.25 µm) with high molecular weight PVP (PVP K30); MPs (0.81–2.43 µm) with low molecular weight PVP (PVP K17)	[53]
	PVP	Curcumin	aggregates, NPs or SMPs with m.s. in the range 0.05–0.33 µm	[54]
	PVP and MCC, starch or lactose	Curcumin	irregular particles/crystals of MCC (size: 175 µm), starch (size: 15 µm) or lactose (size <5 µm) coated with PVP/curcumin particles	[74]
	PVP	Ketoprofen	MPs with m.s. ranging from 2.41 to 3.81 μm	[55]
	PVP	Quercetin and rutin	Quercetin MPs in the range 0.47–9.52 μm and rutin MPs in the range 0.84–8.17 μm	[56]
	PVP	Ezetimibe	NPs with m.s. 0.21–0.23 µm	[43]
	PVP	Oxeglitazar	Crystals	[44]
	PVP	Hydrochlorothiazide	NPs in the range 0.05–0.21 µm	[75]
	PVP	Telmisartan	SMPs and MPs with m.s. 0.38–0.60 µm	[45]
	PVP	Diflunisal	coalescent NPs and coalescent MPs (size in the range 0.4–8.1 µm)	[76]
Co-grinding	PVP/HP-β-CD into chitosan/CaCl_2_/alginate	Celecoxib	MPs	[64]
	PVP	Ingliforib, Furosemide and Celecoxib	stable colloidal particles (m.s. < 370 nm)	[57]
Wet chemical method	PVP	Epirubicin hydrochloride (and iron oxide)	PVP coated NPs with m.s. in the range 60–113 nm	[17]
Free-radical polymerization, solvent evaporation	Amphiphilic PVP	Proteins	Drug-loaded PMs (size: 50–80 nm)	[84]
Free-radical polymerization, solvent evaporation	Amphiphilic PVP	Indomethacin	Drug-loaded PMs (m.s. < 200 nm)	[83]
Ring-opening polymerization, freeze-drying	PVP-b-PDLLA diblock copolymers	Paclitaxel docetaxel	Drug-loaded PMs (size: 20–60 nm)	[87]
Ring-opening polymerization, freeze-drying	PVP-b-PDLLA diblock copolymers	Indomethacin	Drug-loaded PMs (size: 40–100 nm)	[82]
Emulsification or ultrasonic dispersion	Amphiphilic PVP	Curcumin	Drug-loaded PMs (size < 100 nm with dispersion; size: 200–300 nm with the emulsion)	[85]
Dynamic stirring, quenching	PVP	Doxorubicin (and gold)	NPs (m.s. about 14 nm)	[16]

**Table 2:** PVP-based fibers. FBs, fibers; PLLA, poly(l-lactic acid); PCL, polycaprolactone; H-β-CD, hydroxypropyl-β-cyclodextrin; β-CD, β-cyclodextrin; PLGA, polylactic-co-glycolic acid; PLA, polylactic acid; GO, graphene oxide; PPy/I, polypyrrole/iodine.

Technique	Polymeric Carrier	Active Compound	Results	Reference
Electrospinning	PVP	Indomethacin	- complete drug release in about 50 min - easy deposition of FBs onto usual existing dressings	[4]
	PVP	Emodin	- complete drug release after 120 min - accelerated wound healing in 15 days	[23]
	PVP	*Garcinia Mangostana L*. extracts	complete extracts dissolution in 100 min	[24]
	PVP	Ibuprofen	improvement in the disintegration properties	[25]
	PVP	Feruloyl-oleyl-glycerol	improvement in the disintegration properties	[89]
	PVP	Tetracycline hydrochloride	- well-aligned FBs (both as single layer and multilayer) - complete release of antibiotic in 50 min	[95]
	zein/PVP blend	Ketoprofen	complete drug dissolution from 2.5 to 6 h	[102]
	PVP/PLLA blend	Benzoin	sustained benzoin release	[105]
	PVP/PCL blend	*Tecomella undulata* extract	- prolonged release up to 24 h - good bactericidal activity	[101]
	PVP/PCL blend	Trans-anethole	- sustained drug release - promotion of osteoblast differentiation for bone regeneration	[113]
	PVP/PCL blend	-	- FBs loaded with ZnO/Ag nanoparticles - improved antimicrobial activity	[13]
	PVP/HP-β-CD or PVP/β-CD blends	Meloxicam	- improved fibers stability against moisture - improvement in the disintegration properties - complete drug release after 60 min - good masking taste	[106]
	PVP coated with PPy/I	-	- improved viability and adhesion of cells - fast wound healing	[99]
Coaxial electrospinning	*core*: zein *shell*: PVP	Ketoprofen	biphasic drug release: an initial burst (42%) followed by a sustained drug release	[96]
	*core*: PVP *shell*: PLGA	*core*: naringin *shell*: metronidazole	dual release system: a short-term release of metronidazole, a long-term release of naringin	[100]
	*core*: PVP *shell*: PLA	-	- core/shell FBs - no drug incorporated	[104]
Sequential electrospinning	*top/bottom layers*: zein *middle layer*: PVP blended with GO	Ketoprofen	- trilayer FBs - biphasic drug release: an initial burst (60%) from PVP layer in 1 h, followed by a sustained release in 15 h	[103]

**Table 3:** PVP-based hydrogels. HGs, hydrogels; PEG, polyethylene glycol; CA, crotonic acid; BSA, bovine serum albumin; CMC, carboxymethyl cellulose.

Technique	Polymeric Carrier	Active Compound	Results	Reference
Casting	PVP/pectin	Salicylic acid	slightly faster drug release at slightly basic pH	[26]
Crosslinking by electron beam and gamma radiation	PVP/PEG PVP/Laponite	-	- high water uptake, improved elasticity and mechanical properties by adding Laponite - no active principle was loaded	[27]
Grafting by gamma irradiation	PVP grafted with CA	Ketoprofen	targeted release: a low drug release at acid pH compared to neutral/slightly basic pH	[28]
Casting, followed by freeze-drying or air-drying	PVP/chitosan	Amoxicillin	the best drug release achieved in an acid environment	[29]
Electrospinning, followed by crosslinking with UV-C radiation and Fenton reaction	PVP	BSA or collagenase	- high porosity of HGs - improvement in the protein dissolution rate	[5]
Gamma irradiation	PVP/PEG/ agar/CMC	-	- HGs loaded with silver nanoparticles - high antimicrobial activity - accelerated wound healing	[15]
Solution casting, followed by liquid diffusion technique	Biomineralized (CaCO_3_) PVP/CMC	-	- HGs responded to different stimuli: pH and temperature and simulated biological solutions	[131]

**Table 4:** PVP-based tablets. TBs, tablets; VAc, vinyl acetate; CC, cross-carmellose; HPMC, hydroxypropyl methylcellulose; MCC, microcrystalline cellulose; EC, ethyl cellulose; HPC, hydroxypropylcellulose; TEC, triethyl citrate; ERL, Eudragit RL; PVAc, poly(vinyl acetate); PEG, polyethylene glycol.

Technique	Polymeric Carrier	Active Compound	Results	Reference
Spray-drying or ball-milling followed by compression	PVP-VAc	Tadalafil	improved drug dissolution with TBs based on both spray-dried and ball-milled dispersions	[30]
Direct compression	PVP with CC, HPMC, lactose and mannitol	Diclofenac sodium	- good disintegration properties - increase in drug dissolution - the best formulation contained PVP/CC 2/1 *w*/*w*	[134]
*core*: direct compression *coating*: wet granulation	*core*: MCC/CC *coating*: PVP/EC/HPC	Montelukast sodium	the lag time in the release profiles was affected by the PVP content	[31]
*core*: wet granulation followed by compression *coating*: dip or spray coating	*core*: PVP/dextrose/MCC *coating*: PVP/TEC/cellulose acetate	Ketoprofen	PVP K30 was suitable to reach a 24 h drug release	[136]
Both for *core* and *coating*: solvent evaporation followed by compression	*core*: PVP *coating*: PVP/HPMC	Felodipine	drug release profiles with a delay time	[32]
Double compression	*coating* PVP/ERL/NaHCO_3_	5-fluorouracil	PVP/ERL/NaHCO_3_ 68/17/15 *w*/*w*/*w* was the best formulation in terms of floating, mechanical strength, and drug release	[137]
Direct compression	PVP/PVAc	Diprophylline	a drug release model was proposed to facilitate the development of TBs in terms of time and costs	[144]
Supercritical impregnation followed by compression	PVP	Piroxicam	the best release profiles with PVP K15/piroxicam TBs containing less than 13% of drug	[135]
3D printing	PVP	Dipyridamole or theophylline	- good mechanical and disintegration properties - acceptable in-batch variability	[138]
3D printing	PVP	Pantoprazole sodium	PVP TBs allowed a faster drug release compared to other polymers (PEG, poloxamer 407)	[139]

**Table 5:** PVP-based films. NaCMC, carboxymethylcellulose sodium salt; HPMC, hydroxypropyl methylcellulose; PVA, polyvinyl alcohol; EC, ethyl cellulose; DBP, dibutyl phthalate; ERS100, Eudragit RS100; ERSPM, Eudragit RSPM.

Technique	Polymeric Carrier	Active Compound	Results	Reference
Solution casting	PVP	Fentanyl	- good mucoadhesion property - improvement in drug dissolution	[6]
	PVP/NaCMC PVP/HPMC	Ibuprofen	higher performance of PVP films compared to Eudragits films	[33]
	PVP/PVA	Diclofenac sodium	PVP increased the swelling, but it reduced the strength and the elasticity of films	[147]
	PVP/HPMC PVP/EC	Captopril	EC/PVP 3/1 *w*/*v* with 5% of DBP (plasticizer and permeation enhancer) was indicated as the best formulation in terms of drug release, but no release kinetics was shown	[34]
	PVP/EC	Haloperidol lactate	improvement in the drug release	[35]
	PVP/EC	Diltiazem hydrochloride	- PVP/EC 1/2 *w*/*w* was one of the best formulations for a sustained drug release	[36]
	PVP/EC	Diltiazem hydrochloride and indomethacin	- improvement in the drug dissolution and its skin permeation - the release rate was independent of the film thickness - EC/PVP 4/1 *w*/*w* was the best formulation for a controlled drug release	[148]
	PVP/EC PVP/ERS100 PVP/ERSPM	Lornoxicam	- improvement in the drug dissolution and its skin permeation - the best patch consisted of PVP/EC 1.6/1 *w*/*w* + 10% of oleic acid as a plasticizer	[149]
	PVP/rosin	Diltiazem hydrochloride	improvement in the drug dissolution and its skin permeation	[98]
	PVP/guar gum	Diclofenac potassium	improvement in the drug release rate	[150]
